# iTRAQ-based Quantitative Proteomics Analysis Identifies Host Pathways Modulated during *Toxoplasma gondii* Infection in Swine

**DOI:** 10.3390/microorganisms8040518

**Published:** 2020-04-05

**Authors:** Jun-Jun He, Jun Ma, Jin-Lei Wang, Fu-Kai Zhang, Jie-Xi Li, Bin-Tao Zhai, Hany M. Elsheikha, Xing-Quan Zhu

**Affiliations:** 1 State Key Laboratory of Veterinary Etiological Biology, Key Laboratory of Veterinary Parasitology of Gansu Province, Lanzhou Veterinary Research Institute, Chinese Academy of Agricultural Sciences, Lanzhou 730046, China; hejunjun617@163.com (J.-J.H.); dreamerjm@163.com (J.M.); wangjinlei90@126.com (J.-L.W.); kid372820378@163.com (F.-K.Z.); 18088308145@163.com (J.-X.L.); zhaibintao@163.com (B.-T.Z.); 2 Faculty of Medicine and Health Sciences, School of Veterinary Medicine and Science, University of Nottingham, Sutton Bonington Campus, Loughborough LE12 5RD, UK

**Keywords:** *Toxoplasma gondii*, pigs, proteome, immune proteins, co-expression

## Abstract

*Toxoplasma gondii* is a leading cause of foodborne illness and consumption of undercooked pig meat is a major risk factor for acquiring toxoplasmosis, which causes a substantial burden on society. Here, we used isobaric tags for relative and absolute quantification (iTRAQ) labelling coupled with liquid chromatography-tandem mass spectrometry (LC-MS/MS) to identify cellular proteins and pathways altered during *T. gondii* infection in pigs. We also used parallel reaction monitoring-based LC-MS/MS to verify the levels of protein expression of infected spleens and mesenteric lymph nodes (MLNs). At 6 days post-infection (dpi), 156, 391, 170, 292, and 200 differentially expressed proteins (DEPs) were detected in the brain, liver, lung, MLNs and spleen, respectively. At 18 dpi, 339, 351, 483, 388, and 303 DEPs were detected in the brain, liver, lung, MLNs and spleen, respectively. Although proteins involved in immune responses were upregulated in all infected tissues, protein expression signature in infected livers was dominated by downregulation of the metabolic processes. By weighted gene co-expression network analysis, we could further show that all proteins were clustered into 25 co-expression modules and that the pink module significantly correlated with the infection status. We also identified 163 potential anti-*T. gondii* proteins (PATPs) and provided evidence that two PATPs (HSP70.2 and PDIA3) can reduce *T. gondii* burden in porcine macrophages in vitro. This comprehensive proteomics analysis reveals new facets in the pathogenesis of *T. gondii* infection and identifies key proteins that may contribute to the pig’s defense against this infection.

## 1. Introduction

Toxoplasmosis, a widespread zoonosis, is caused by the opportunistic protozoan parasite *Toxoplasma gondii.* This parasite has a remarkable ability to infect almost all warm-blooded vertebrates [1]. Humans and animals acquire *T. gondii* through ingestion of undercooked or raw meat containing bradyzoites within cysts or drinking water contaminated with sporulated oocysts. Tachyzoite is another infective stage of *T. gondii* and differentiates into bradyzoite at 10–14 days post infection [2]. *T. gondii* infection is often benign in immunocompetent individuals. However, reactivation of latent infection in immunocompromised patients can cause brain abscesses, encephalitis and chorioretinitis, and primary infection during pregnancy can lead to abortion or severe health consequences (e.g. ocular impairment and neurological sequelae) in prenatally affected children [3]. The presence of *T. gondii* cysts in the brain has also been associated with altered predator aversion of murine hosts toward cats [4,5], and neuropsychological conditions, such as schizophrenia in humans [6,7,8]. Chronic *T. gondii* infection can even alter subpopulations of neurons in pigs [9].

Although mice have been used as a powerful model to investigate the molecular pathophysiology of toxoplasmosis, biological differences exist between mice and humans, in terms of susceptibility to and immune response against *T. gondii* infection [10,11,12]. Pigs are anatomically and physiologically similar to humans [13], and their susceptibility to infection is more similar than that of mice [14]. Therefore, a pig model of infection would be relevant for investigating the determinants of *T. gondii* pathogenesis. The significant role of pigs in the transmission of *T. gondii* infection to humans [15,16] is another reason to understand the mechanism of toxoplasmosis disease in this animal species.

*T. gondii* has the capability to coordinate host gene expression and evade several immune defense mechanisms in order to establish acute or chronic infection within the host [17,18,19,20]. Proteomic analysis has also revealed many host proteins that play a role in the mouse response to *T. gondii* infection [17]. The proteome links phenotype to genotype and its investigation can reveal many molecular events that influence host cellular mechanisms during infection [21]. However, how the *T. gondii* infection reshapes protein expression of porcine tissues during *T. gondii* infection remains largely unknown.

In the present study, we used a quantitative proteomics approach to investigate the effect of *T. gondii* infection on the proteome of multiple tissues of pigs. We employed tandem mass spectrometry because it enables a global understanding of the function and regulation of the proteome [22]. Bioinformatics analysis was performed to identify the differentially expressed proteins (DEPs), which can play a role in the pathophysiology of *T. gondii* infection. Additionally, seven DEPs were selected for parallel reaction monitoring (PRM) analysis in order to verify the expression results obtained by mass spectrometry analysis.

## 2. Materials and Methods

### 2.1. Animals, Parasite Challenge, Sample Collection and T. gondii Detection

Specific-pathogen-free (SPF) white pigs were purchased from Beijing Center for SPF Swine Breeding and Management. All pigs were handled strictly in accordance with the Animal Ethics’ Procedures and Guidelines of the People’s Republic of China. The study design was reviewed and approved by the Animal Ethics Committee of Lanzhou Veterinary Research Institute, Chinese Academy of Agricultural Sciences. The sera of all pigs were screened for anti-*T. gondii* antibodies using a modified agglutination test (MAT). The pigs used in the study were seronegative for *T. gondii*.

The study included 24 pigs divided into eight groups (3 pigs/group): 6C_1 (Control group slaughtered at 6 days post infection [dpi], replicate 1), 6C_2 (Control group slaughtered at 6 dpi, replicate 2), 18C_1 (Control group slaughtered at 18 dpi, replicate 1), 18C_2 (Control group slaughtered at 18 dpi, replicate 2), 6T_1 (Treatment group slaughtered at 6 dpi, replicate 1), 6T_2 (Treatment group slaughtered at 6 dpi, replicate 2), 18T_1 (Treatment group slaughtered at 18d dpi, replicate 1), and 18T_2 (Treatment group slaughtered at 18 dpi, replicate 2). In the infected groups, each pig was inoculated orally with 1000 oocysts of *T. gondii* PYS strain (genotype ToxoDB#9) mixed with 100 g pig feed in 5 mL sterile phosphate-buffered saline (PBS). Each pig in the control groups was mock inoculated with 100 g pig feed mixed with 5 mL sterile PBS. Brains (cerebral cortices), livers, spleens, lungs and mesenteric lymph nodes (MLNs) of pigs were collected at 6 and 18 dpi. The collected tissues were stored frozen at −80 °C until used for proteomic analysis and DNA extraction.

For in vitro experiment, 3D4/2 cell lines were challenged with *T. gondii* tachyzoites at multiplicity of infection (MOI) of 1. After 48 h, the infected cells were collected, and their DNAs were extracted for detection of relative *T. gondii* load. Genomic DNA was extracted using TIANamp Genomic DNA Kit (Tiangen Biotech, Beijing, China) according to the manufacturer’s instructions and stored frozen at −20 °C until use. The *T. gondii B1* gene was used for *T. gondii* detection and quantification, and the pig *18S* rRNA gene was used to normalize *T. gondii* B1 DNA to host DNA. The primers used for *T. gondii B1* gene detection/quantification were: B1F: TGCATAGGTTGCAGTCACTG and B1R: TCTTTAAAGCGTTCGTGGTC (the expected product size is 131 bp). The primers used for pig DNA normalization were: 18S-pigF: GCCTGCTGCCTTCCTTG and 18S-pig: ATGGTAGTCGCCGTGCC (the expected product size is 109 bp). Quantitative real time polymerase chain reaction (q-PCR) was performed on the Rotor-Gene Q (QIAGEN, Hilden, Germany) using SYBR Green GoTaq qPCR Master Mix (Promega, Beijing, China) according to the manufacturer’s instructions. SYBR Green q-PCR cycling conditions included 95 °C for 5 min followed by 50 cycles of 95 °C for 10 s, 60 °C for 10 s, and 72 °C for 15 s. The temperature of melting curve analysis ranged from 72 °C to 95 °C to ensure specificity of the qPCR products. The 2^-ΔΔCT^ relative expression method was used for the calculation of the relative *T. gondii* load [23].

### 2.2. Protein Extraction and iTRAQ Analysis

Protein extraction and iTRAQ labeling were performed as previously described [17]. Briefly, pig tissues were individually ground into powder using liquid nitrogen, and protein was extracted with lysis buffer (4% CHAPS, 7M Urea, 1 mM PMSF, 40 mM Tris-HCl, 2M Thiourea, 2 mM EDTA, pH 8.5). Protein quantification was measured by using Bradford assay [24]. The extracted proteins were kept at −80 °C until used for iTRAQ analysis. Before iTRAQ labeling, an equal amount of protein from each sample of the same group were pooled as one replicate. For each pooled replicate, ~100 μg protein was digested with Trypsin Gold (Promega, Madison, WI, USA) at an enzyme-protein ratio of 1:30 (w/w) at 37 °C for 16 h. The resulting peptides were dried by vacuum centrifugation and reconstituted in 0.5 M triethyl ammonium bicarbonate (TEAB) (Thermo Fisher Scientific, Inc.) and processed according to the manufacture’s protocol for 8-plex iTRAQ reagent (Applied Biosystems). SCX chromatography was performed with a LC-20AB HPLC Pump system (Shimadzu, Kyoto, Japan). Peptides elution was monitored for absorbance at 214 nm, and fractions were collected every 1 min. The eluted peptides of the same replicate were divided into 20 fractions and desalted with a Strata X C18 column (Phenomenex) and then vacuum-dried. Each fraction was analyzed using LC-ESI-MS/MS based on Triple TOF 5600 System (AB SCIEX, Concord, ON) fitted with a Nanospray III source (AB SCIEX, Concord, ON) and a pulled quartz tip as the emitter (New Objectives, Woburn, MA). Protein identification was achieved using commercial Mascot search engine (Matrix Science, London, UK; version 2.3.02) against Uniprot database and two unique peptides were required for protein identification. Student’s *t*-test was used for significant expressional change analysis. The resulting dataset was auto bias-corrected to the biological replicate, and quantitative protein ratios were weighted and normalized by the median ratio using the Mascot software. *p*-values <0.05 and fold change >1.2 (upregulated) or <0.83 (downregulated) were used as cut-off to determine proteins with significantly altered expression levels. The protein extraction, iTRAQ analysis, protein identification and quantification were performed at BGI-Shenzhen, China.

### 2.3. Targeted Protein Quantification by Parallel Reaction Monitoring (PRM)

iTRAQ-based results were validated using a PRM-based LC-MS/MS approach. Seven proteins were selected for verification, including HSP90AA1, OXCT1, ARL6IP5, GBP7, GBP1, PDIA3 and GVIN2. The proteins of spleen and MLN samples were separately extracted as described above. The isolated proteins were used for PRM analysis. Briefly, the extracted proteins were incubated with 10 mM dithiothreitol (DTT) (Thermo Fisher Scientific, Inc.) at 56 °C for 30 min, followed by incubation with 55 mM iodoacetamide (IAM) (Promega, Madison, WI, USA) for 30 min and addition of 4-fold volume chilled acetone and incubation at −20 °C overnight. This was followed by centrifugation of the mixture and discarding of the supernatant. The protein was dissolved with 8 M urea/100 mM TEAB (pH 8.0) (Thermo Fisher Scientific, Inc.) and quantified using the Bradford assay. Then, 100 μg protein were digested with trypsin overnight at 37 °C and the protein digestion was terminated by trifluoroacetic acid (TFA). The peptides were desalted by C18 column (Phenomenex) and vacuum dried. The dried peptides were subjected to LC-ESI-MS/MS analysis based on a Triple TOF 5600 System (AB SCIEX, Concord, ON), and the PRM data were collected. The PRM experiments and data analysis were completed by Wuhan GeneCreate Biological Engineering Co., Ltd.

### 2.4. Bioinformatics Analysis of Proteins

KOBAS3.0 [25] was used for Kyoto Encyclopedia of Genes and Genomes database (KEGG) pathway enrichment analysis. Fisher’s exact test was performed to evaluate the level of significance in the enrichment analysis. Gene Ontology (GO) enrichment analysis was performed with the threshold of *p* < 0.05 for three categories biological process, molecular function, and cellular component. *p*-value calculated by Fisher’s exact test was further adjusted by false discovery rate (FDR) correction [26]. FDR corrected *p*-values under a threshold of 0.05 were considered significant. WGCNA R package [27] was used for protein co-expression and trait correlation analyses. In WGCNA analysis, normalized expression of all proteins and the infection status were used as input data. The soft power detection ranged from 1 to 20 and the lowest power at which the scale-free topology fit index reaches 0.8 was chosen as the soft power that was used for constructing a weighted gene co-expression network. Infection status (infected samples were assigned 1 and control samples were assigned 0) was used as a correlative trait. For testing the predictive performance between the protein expression level and *T. gondii* infection, pROC R package [28] was applied to analyze the receiver operating characteristic curve (ROC) and area under curve (AUC) between the module genes and *T. gondii* infection. Proteins interaction network was analyzed using STRING database [29].

### 2.5. Construction of HSP70.2- and PDIA3-Expressing Plasmids and Transfection of 3D4/2 Cells

The pig CRISPR-Cas9 plasmid was constructed as shown in Supplementary Figure S1A and was designed with sgRNA sequence (GTTCCTGGAAGTTTAGATCA) that targets the *H11* gene locus. *H11* is one of the pig genome loci known as a safe harbor, in which an exogenous gene can be inserted while circumventing potential “positional effects” and avoiding the interference in the genome. Gene over-expression donor scaffold (H11-cmv-c-HA over-expression donor plasmid) was synthesized by Genewiz (Genewiz, China). The complete sequence and sequence annotations of the donor plasmid are shown in Supplementary File S1. The primers used for gene cloned into H11-cmv-c-HA over-expression donor plasmid and knock-in detection are listed in Supplementary Table S1. The HSP70.2-expressing plasmids were constructed as follows: the full-length (1923 bp) cDNA of target gene (*HSP70.2*) was amplified from RNA extracted from the tissue of a healthy pig using RT-PCR with primers HSP70.2-F (5’- GCGGTGGCGGCCGCTCTAGGCCACCATGGCGAAGAGCGTGGCCATCGGCAT-3’) and HSP70.2-R (5’- ATCTGGAACATCGTATGGGTAATCCACCTCCTCGATGGTGGGGC-3’). Likewise, *PDIA3* coding sequence was amplified using primers PDIA3-F (5’- GCGGTGGCGGCCGCTCTAGGCCACCATGCGCCTCTGCCGCCTAGCGCT-3’) and PDIA3-R (5’-ATCTGGAACATCGTATGGGTAGAGATCCTCCTGTGCCTTCTTCT-3’). The PCR products were ligated with knock-in donor fragment using ClonExpress MultiS One Step Cloning Kit (Vazyme Biotech Co., Ltd, China) according to the manufacturer’s instructions. Knock-in donor fragment was amplified from H11-cmv-c-HA donor plasmid using primers Vector-F (5’-TACCCATACGATGTTCCAGAT-3’) and Vector-R (5’-GGTGGCCTAGAGCGGCCGCCACCGC-3’). The plasmid that was successfully constructed with target gene CDS and knock-in donor fragment was extracted using Endofree Maxi Plasmid Kit (TIANGEN, China), and digested using NruI enzyme for linearization of knock-in donor (named as transfection donor in Supplementary Figure S1B). The pig CRISPR-Cas9 plasmid and linearized knock-in donor were co-transfected into porcine macrophage cell line 3D4/2 (ATCC, CRL-2845) using Xfect^TM^ Transfection Reagent (Takara, China). Forty-eight hours post transfection, transfected cells were selected with 2 μg/mL puromycin, 10% fetal bovine serum (FBS), 90% high glucose DMEM/F12 for 4 days. The puromycin-resistant cells were plated in limiting dilution in 96-well plates for isolation of single clones. The genomic knock-in was validated by PCR using primers Knock-in-F and Knock-in-R (Supplementary Table S1).

### 2.6. Validation of the Expressed Proteins

Western blotting analysis was performed to examine whether knock-in genes were correctly expressed in the 3D4/2 cells. The total proteins were extracted from transfected cells using ProteinExt® Mammalian Total Protein Extraction Kit (Transgene, China). Approximately 30 μg total protein and 10 μL Blue plus IV Protein Marker (Transgen, China) were loaded on 12% Expressplus^TM^ PAGE Gels (GenScript, China) and electrophoresis was run at 100V for 1.5 h. The proteins were electrically transferred to PVDF membrane (Thermo, Germany) at 20V for 30 min using Trans-Blot SD apparatus (Bio-Rad, USA), and then blocked with 5% fat-free milk for 1 h. The PVDF membrane was incubated with primary anti-HA tag antibody (Abcam, UK) at 4 °C overnight and then washed three times with tris-buffered saline (TBS). The PVDF membrane was then incubated with goat anti-mouse IgG H&L (HRP) antibody (Abcam, UK) at 37 °C for 1 h. The PVDF membrane was washed three times using TBS buffer and incubated with the ECL reagent (Solarbio, China) for chemiluminescence detection. The images were obtained by X-ray film (Carestream, China).

We also performed immunofluorescence analysis of the over-expressed proteins. Wild type 3D4/2 cells and protein over-expressing cells were grown on coverslips and challenged with *T. gondii* at multiplicity of infection (MOI) of 1. After 48 h, infected cells were washed three times with PBS and fixed with 4% paraformaldehyde in PBS (Solarbio, China) for 10 min. The fixed cells were washed with PBS three times and permeabilized with 0.1% TritonX-100 (Beyotime, China) for 20 min. The cells were blocked with 5% bovine serum albumin for 1 h and washed with PBS for three times. The cells were incubated with primary mouse anti-HA tag antibody (Abcam, UK) overnight at 4 °C. The cells were washed three times with PBS and the parasites were detected with primary anti-*T. gondii* antibody (FITC) (Abcam, UK), followed by goat anti-mouse IgG H&L secondary antibody, conjugated with Alexa Fluor^®^647 (Abcam, UK) at 37 °C for 2 h. The cell nucleus was stained with 100 ng/mL DAPI (Invitrogen) for 10 min. The coverslips were placed on a glass slide in mounting medium (Solarbio, China) and examined with a confocal laser scanning microscopy platform Leica TCS SP8 (Leica, Germany).

## 3. Results

### 3.1. Confirmation of T. gondii Infection

All pigs in the infected groups showed clinical signs at 6 dpi, such as fever and inappetence, whereas pigs in the control groups remained apparently healthy. PCR results showed that all collected tissues of infected groups were *T. gondii B1* gene positive. However, the tissues of control groups were *B1* gene negative.

### 3.2. Proteomic Features of Infected Tissues

**Figure 1:**
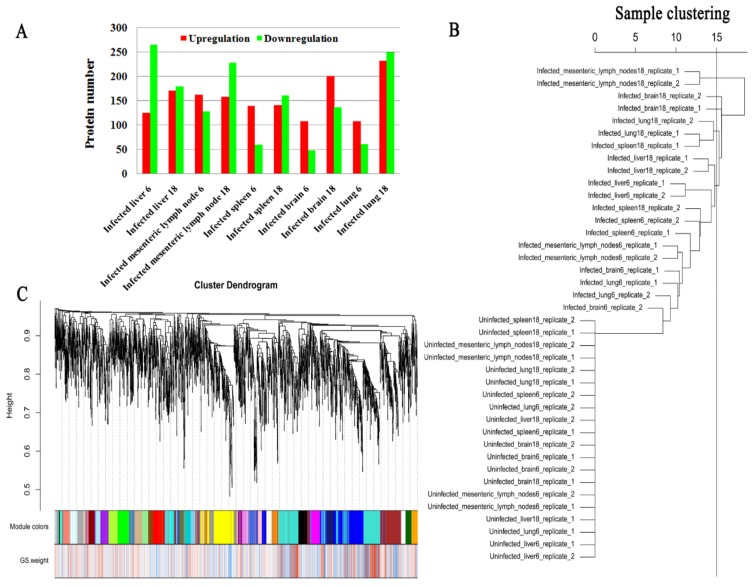
Summary of the differentially expressed proteins (DEPs) and co-expression analysis of protein expression profile. (**A**) The number of upregulated and downregulated proteins in each pig tissue. (**B**) Sample clustering based on their protein profiles. (**C**) Cluster dendrogram of protein. GS weight denotes gene significant weight.

Our analysis revealed 4706, 4224, 4535, 5103 and 4414 proteins in pig brain (cerebral cortex), liver, lung, MLNs and spleen, respectively. More than 300,000 spectra were obtained in each tissue. The summary of protein identification data is shown in Supplementary Table S2. Most mean variation coefficients (CV) were <0.1, and >90% of the proteins had CV < 0.3, indicating that our iTRAQ analysis had good repeatability and performance (Supplementary Table S3). At 6 dpi, 108, 125, 109, 163, and 140 DEPs were upregulated, whereas 48, 266, 61, 129, and 60 DEPs were downregulated in infected brain, liver, lung, MLNs and spleen, respectively. At 18 dpi, 202, 171, 233, 159, and 141 DEPs were upregulated, but 137, 180, 250, 229, and 162 DEPs were downregulated in infected brain, liver, lung, MLNs and spleen, respectively (Figure 1A). No common DEP was found among the tissue groups examined (Supplementary Figure S2). To validate the expression data, targeted mass spectrometry analysis using PRM was performed. As shown in Supplementary Figure S3, PRM expression data of seven selected proteins (HSP90AA1, OXCT1, ARL6IP5, GBP7, GBP1, PDIA3 and GVIN2) of spleens and MLNs showed similar expressional trends to iTRAQ-based mass spectrometry data. All mass spectrometry raw data are available at ProteomeXchange database with accession numbers: PXD011816, PXD011828, PXD011829, PXD011830 and PXD011831.

### 3.3. Enrichment Analysis of Differentially Expressed Proteins (DEPs)

GO and KEGG enrichment analyses of the DEPs revealed that *T. gondii* infection can significantly upregulate dozens of biological processes in infected brain at 18 dpi, such as small GTPase mediated signal transduction, actin filament-based process, vesicle-mediated transport and antigen processing and presentation. Only a few pathways were significantly enriched in the brain at 6 dpi. DEPs of vesicle-related GO terms were upregulated in the infected brains (Supplementary File S2). In infected livers, at 6 dpi and 18 dpi, the significantly altered biological processes involved downregulation of the metabolic processes, such as oxidation-reduction process, carboxylic acid metabolic process, oxidoreduction coenzyme metabolic process, pyruvate metabolic process, and tryptophan metabolism. Additionally, upregulation of immune response related processes was also detected in infected liver, especially at 18 dpi (Supplementary File S3). In the infected lung, spleen and MLNs, immune response-related processes/pathways were significantly altered, including upregulation of antigen processing and presentation, chaperone-mediated protein folding, complement and coagulation cascades, response to stress, and innate immune response. The details of KEGG enrichment analysis of DEPs (including GO annotations according to biological processes, cellular components, and molecular functions) in infected lung, spleen and MLNs are listed in Supplementary Files S4–S6, respectively.

### 3.4. Co-Expression and Trait Correlation Analyses

**Figure 2:**
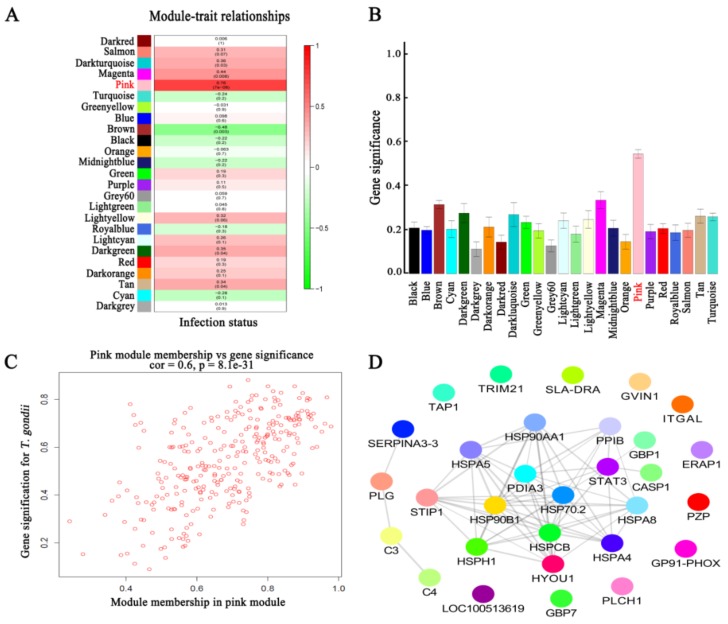
Relationship between the modules and *T. gondii* infection. (**A**) Relationship between modules and *T. gondii* infection status. (**B**) Level of gene significance of the different modules. (**C**) Pearson’s correlation coefficient between gene significance for *T. gondii* and module membership in the pink module. (**D**) Interaction network of the top 30 hub genes.

**Figure 3:**
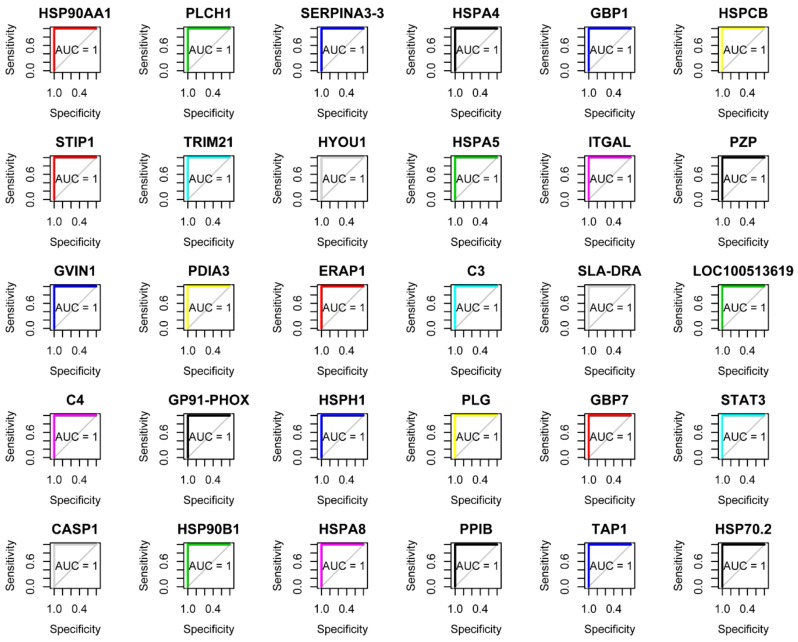
Summary of the receiver operating characteristic (ROC) analysis and associated area under the curve (AUC). ROC curves show the correlation between the top 30 hub proteins and *T. gondii* infection.

**Figure 4:**
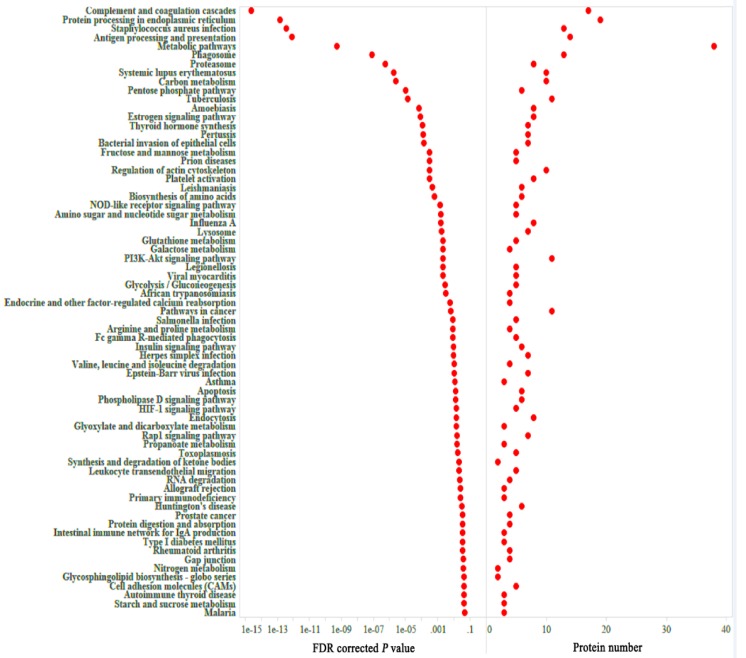
Kyoto Encyclopedia of Genes and Genomes database (KEGG) enrichment analysis of genes in the pink module.

The WGCNA R package was used for protein co-expression and trait correlation analyses. As shown in Figure 1B, infected and non-infected tissues were separated into two main branches according to their respective protein expression patterns. The soft power (3) was the first power value that showed scale free topology model fit value > 0.8 (Supplementary Figure S4A). Additionally, soft power (3) showed a relatively high gene connectivity (Supplementary Figure S4B). Therefore, the number 3 was used as soft power for building the co-expression network. We detected 25 co-expression modules (Figure 1C). The modules, traits and *T. gondii* gene significance relationship are shown in Figure 2A,B. Pink module was significantly correlated with *T. gondii* infection supported by a correlation coefficient of 0.76 and *p*-value of 7 × 10^−8^. The Pearson correlation coefficient between gene significance and gene module membership in the pink module was 0.6 with *p*-value 8.1 × 10^−31^ (Figure 2C). Pink module contained 301 proteins. Top 30 proteins that showed the highest gene significant for *T. gondii* infection included: HSP90AA1, PLCH1, SERPINA3-3, HSPA4, GBP1, HSPCB, STIP1, TRIM21, HYOU1, HSPA5, ITGAL, PZP, GVIN1, PDIA3, ERAP1, C3, SLA-DRA, LOC100513619, C4, GP91-PHOX, HSPH1, PLG, GBP7, STAT3, CASP1, HSP90B1, HSPA8, PPIB, TAP1 and HSP70.2. The interaction network of the top 30 genes is shown in Figure 2D. All top 30 genes showed excellent performance to predict *T. gondii* infection as shown by AUC of all these genes (Figure 3). Table 1 shows the orthology description of the top 30 genes. KEGG enrichment analysis of the genes in the pink module revealed that 69 pathways were significantly enriched, such as complement and coagulation cascades, protein processing in endoplasmic reticulum, antigen processing and presentation, phagosome, proteasome and NOD-like receptor signaling pathway (Figure 4).

### 3.5. Identification and Functional Analysis of Potential Anti-T. gondii Proteins (PATPs)

**Figure 5:**
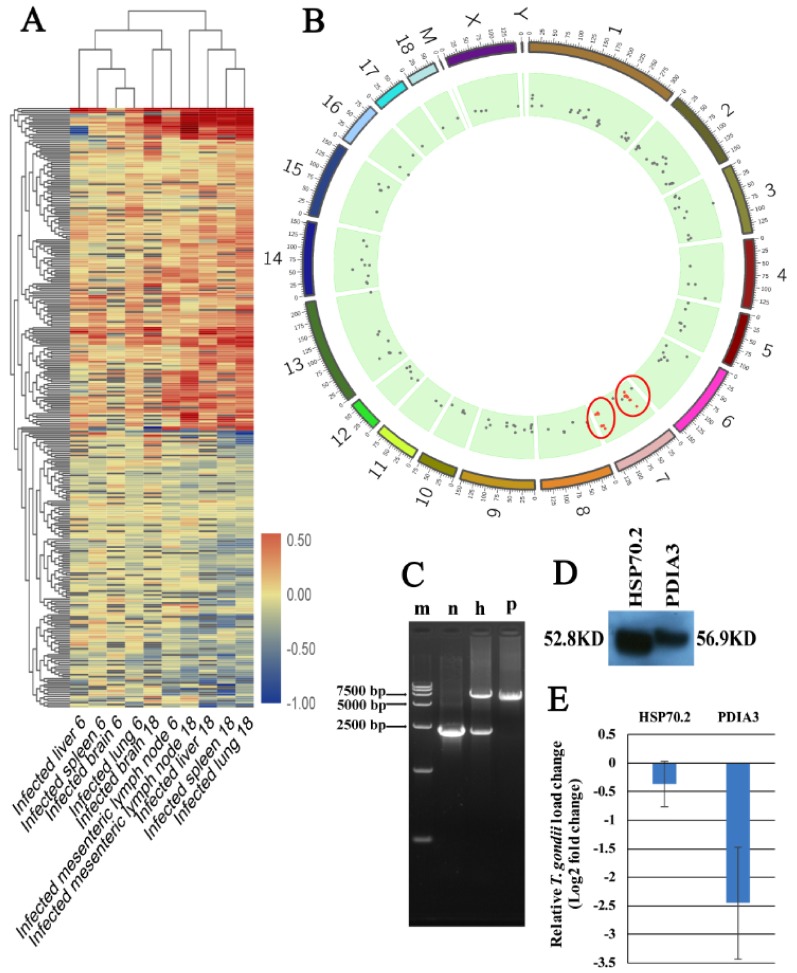
Clustering of genes in the pink module and the potential anti-*T. gondii* proteins (PATPs). (**A**) Clustering of the genes in the pink module. (**B**) Genomic distribution of PATPs. Red circles enclose hotspots of the chromosome 7. (**C**) Polymerase chain reaction (PCR) detection of *H11* locus of HSP70.2 or PDIA3 over-expressing elements. Abbreviations: m: DNA marker DL15000; n: wild type 3D4/2 cell line; h, HSP70.2 over-expressing cell line; p, PDIA3 over-expressing cell line. (**D**) Western blot analysis of HSP70.2 and PDIA3 in the two over-expressing cell lines. (**E**) Relative *T. gondii* load in the two over-expressing cell lines.

**Figure 6:**
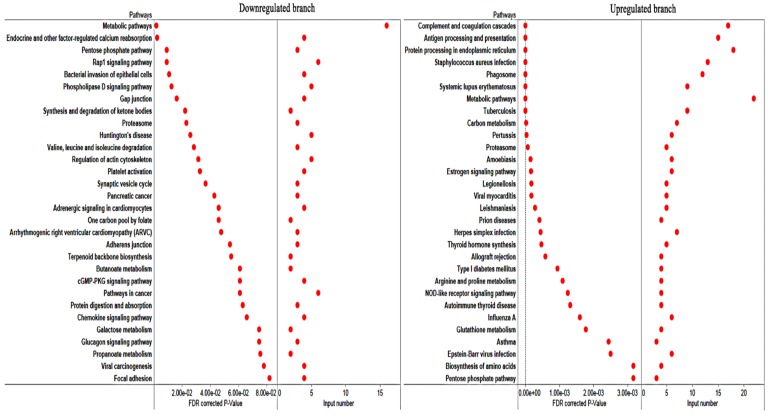
KEGG enrichment analysis of the genes in the two branches of the pink module.

**Figure 7:**
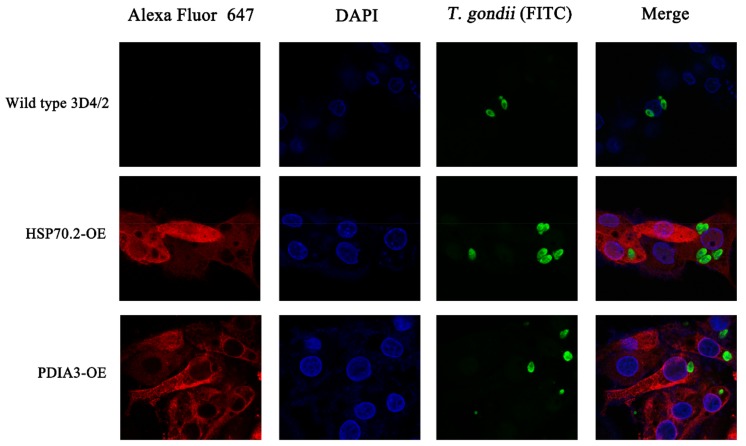
Immunofluorescence analysis of transgenic 3D4/2 cells stably overexpressing (OE) *HSP70.2* and *PDIA3* genes compared to control (wild type) cells. Images show 3D4/2 cells expressing high levels of HSP70.2 and PDIA3 proteins.

The pink module was highly correlated with *T. gondii* infection. It is sensible to anticipate that the gene that has an anti-*T. gondii* property should be highly correlated with *T. gondii* infection and upregulated in infected tissues. As shown in Figure 5A, the proteins in the pink module were categorized into upregulated branch and downregulated branch. Therefore, the genes in the upregulated branch were identified as PATPs that could contribute to anti-*T. gondii* processes. Details of these PATPs are listed in Supplementary File S7. By analyzing the gene locations of PATPs, we found that chromosome 7 has two *T. gondii* hotspots that showed high density of PATPs (Figure 5B, Table 2). Most genes in the two hotspots participate in antigen presentation or other immune-related pathways (Table 2). Two hub proteins of the top 30 genes (Figure 2D) were identified as PATPs (HSP70.2 and PDIA3) because they were highly expressed and were thus selected for functional validation. PCR amplification of porcine *H11* locus showed that, as expected, PDIA3 and HSP70.2 over-expression cassette was successfully inserted into *H11* locus as shown by a fragment size of about 6,400–6,800 bp, while wild type 3D4/2 cell line showed normal size (1,878 bp) (Figure 5C). The PCR product size was consistent with the expected size, suggesting that HSP70.2 or PDIA3 over-expression element was correctly inserted into *H11* locus. However, HSP70.2 over-expression element was only inserted into one of the two allelic *H11* locus. Western blotting result showed that the product of HSP70.2 over-expressing cells was about 52.8 KD, and the product of PDIA3 over-expressing cells was about 56.9 KD. The over-expressed HSP70.2 and PDIA3 proteins are consistent with the predicted sizes (Figure 5D). The two protein over-expressing cell lines showed lower *T. gondii* loads compared with wild type 3D4/2 macrophages (Figure 5E). Pathway enrichment analysis was performed to analyze the functions of the significantly enriched genes in the downregulated (139 proteins) and the upregulated branch (163 proteins). As shown in Figure 6, genes in the downregulated branch were enriched in metabolism, cell migration, cell interaction and other related pathways. However, the genes in the upregulated branch were enriched in immune response, pathogen infection and other related pathways. The immunofluorescence analysis results also confirmed that HSP70.2 and PDIA3 were successfully expressed in the 3D4/2 cells (Figure 7).

## 4. Discussion

The present study is the first to provide a comparative analysis of the proteomic response of five swine tissues to *T. gondii* infection using iTRAQ-based quantitative proteomic approach. Our analysis identified many altered cellular pathways and DEPs during *T. gondii* infection. To verify the results obtained by mass spectrometry analysis, expression profiles of seven proteins in infected spleens and MLNs, including four PATPs (HSP90AA1, GBP1, PDIA3 and GBP7) and three non-PATPs (OXCT1, ARL6IP5 and GVIN2), were examined using targeted PRM analysis. As shown in Supplementary Figure S3, the expression profiles of the seven proteins were similar to the expression profiles obtained by iTRAQ analysis, supporting the validity of the protein expression data.

GO and KEGG pathway analyses have shown that vesicle related processes were upregulated in most infected tissues, including brain, spleen, lung and MLNs (Supplementary Files S2–S6). However, vesicle-related processes were downregulated in infected liver at 6 dpi (Supplementary File S3), which disagrees with upregulation of the vesicle-related processes detected previously in the liver of infected mice [17,44]. However, downregulation of the metabolic process of infected pig liver in the present study was also observed in the mouse model [17,44]. About 190 proteins, involved in metabolic processes, were differentially expressed in the infected livers, most of them downregulated (Supplementary File S3). This suggests that downregulation of the metabolism could be ubiquitous in the liver during *T. gondii* infection. On the contrary, in all infected tissues, immune- or infection-related pathways were upregulated, including upregulation of antigen processing and presentation, chaperone-mediated protein folding, and complement and coagulation cascades. This suggests that anti-*T. gondii* mechanisms were activated and some of these could be common between different tissues and important for helping the swine host to counter *T. gondii in vivo*.

Although we found >100 DEPs in infected tissues (Figure 1A), after intersecting the DEP sets across tissues, there was not any DEP shared across all tissues as shown in the vertical bars and the connected black circles below the histogram (Supplementary Figure S2). Co-expression and trait correlation analysis can reveal common host pathways that may contribute to countering *T. gondii* infection. As shown in Figure 1B, the protein profiles of infected tissues were different from non-infected tissues, indicating that biological responses of infected tissues have been altered by *T. gondii* infection. According to the protein profile cluster dendrogram, 25 protein co-expression modules were found (Figure 1C). Regarding correlation with infection status, we found that the pink module showed the highest correlation with *T. gondii* infection. Global correlation coefficient between *T. gondii* infection and pink module protein profile was 0.76 with *p* value 7 × 10^−8^ (Figure 2A). A high gene significance was also detected in pink module protein (Figure 2B,C). As shown in Figure 2C, the global correlation coefficient between gene module membership and gene significant for *T. gondii* infection was 0.6 with the *p* value 8.1 × 10^−31^. These data indicate that proteins in the pink module are associated with *T. gondii* infection.

We also performed KEGG pathway enrichment analysis in order to analyze the significantly enriched functions of the proteins in the pink module. As shown in Figure 4, the most enriched pathways were infection, disease and immune related pathways, including complement and coagulation cascades, protein processing in endoplasmic reticulum, and antigen processing and presentation. This suggests that most proteins in the pink module can participate in anti-infection processes. The top 30 proteins that showed the highest gene significance for *T. gondii* infection included HSP90AA1, PLCH1, SERPINA3-3, HSPA4, GBP1, HSPCB, STIP1, TRIM21, HYOU1, HSPA5, ITGAL, PZP, GVIN1, PDIA3, ERAP1, C3, SLA-DRA, LOC100513619, C4, GP91-PHOX, HSPH1, PLG, GBP7, STAT3, CASP1, HSP90B1, HSPA8, PPIB, TAP1, and HSP70.2.

ITGAL (also known as CD11A) is expressed on antigen-experienced CD4^+^ and CD8^+^ T cells [41]. GBP1, GBP7, TRIM21, ERAP1, C3, C4, CASP1 and TAP1 have been confirmed as important anti-*T. gondii* factors in mice or humans [31,33,34,35,39,45]. To further confirm whether the top 30 proteins are good predictor of *T. gondii* infection, ROC analysis was performed. This analysis showed that all top 30 proteins are robust markers (AUC = 1) for prediction of *T. gondii* infection in the infected tissues (Figure 3). In general, host anti-*T. gondii* factors are upregulated during *T. gondii* infection. As shown in Figure 5A, the proteins in the pink module were clustered into two branches, including 163 proteins in the upregulated branch and 139 proteins in the downregulated branch. All the top 30 proteins were clustered into the upregulated branch and most of them are protein chaperones involved in protein quality control or MHC peptide process (Table 1). This finding is consistent with the fact that protein quality control and antigen presentation pathways are important for host response against *T. gondii* infection [32,33,39].

We further analyzed the functions of genes in both upregulated and downregulated branches. As shown in Figure 6, the genes of the downregulated branch were significantly enriched in metabolism, cell migration or interaction. However, genes of the upregulated branch were significantly enriched in immune or infection related pathways. Therefore, genes in the upregulated branch were deemed as PATPs. By analyzing the gene distributions on the pig genome, we identified two PATPs hotspots in porcine chromosome 7. Most of the genes distributed in these two hotspots participate in antigen presentation or other immune response related pathways (Table 2).

To determine the function of the identified PATPs, CRISPR-Cas9 method was used for the construction of PDIA3- and HSP70.2-over-expressing cell lines. We selected two PATPs (PDIA3 and HSP70.2) that showed high expression and were the hub proteins of the top 30 proteins, for the construction of over-expressing cell lines. Our PCR and DNA sequencing results showed that PDIA3 and HSP70.2 over-expression cassette was correctly inserted into *H11* locus, which is one of the safe harbors in the pig genome (Figure 5C), although HSP70.2 cassette was only inserted into one *H11* allele. Western blot analysis showed that the HA tagging PDIA3 and HSP70.2 was correctly expressed in the two over-expressing cell lines (Figure 5D). HSP70.2 and PDIA3 are two key proteins in antigen presentation pathway and facilitate the antigen presented by MHC-I complex. As anticipated, HSP70.2 and PDIA3 over-expressing cell lines showed lower *T. gondii* loads compared with wild type 3D4/2 macrophages, particularly the PDIA3 over-expressing cells. Our study also showed that HSP70.2 and PDIA3 can reach *T. gondii* (Figure 7), indicating that HSP70.2 and PDIA3 may facilitate *T. gondii* antigen presentation and thereby contributing to limiting *T. gondii* replication within the host cells. Besides HSP70.2 and PDIA3, we identified many other proteins that could potentially have anti-*T. gondii* properties and can reveal novel mechanisms of host response against *T. gondii* infection *in vivo*.

## 5. Conclusions

The protein expression profile in the brain, liver, lung, spleen and MLNs of pigs during *T. gondii* infection was investigated by iTRAQ technique. This proteomic analysis revealed > 100 DEPs in each infected tissue. Immune responses were active across all infected tissues, whereas downregulated metabolic processes were detected in infected livers, and vesicle mediated transportation was upregulated in infected brains. Using trait correlation analysis, we identified 25 co-expression modules and many PATPs. We also demonstrated that the swine macrophage cells overexpressing two PATPs (HSP70.2 and PDIA3) showed enhanced resistance to *T. gondii* infection. Further elucidation of the functions of other PATPs identified in this study will improve our understanding of how pigs respond to *T. gondii* infection. Ultimately, this work can provide a solid foundation for the development of disease-resistant pigs which could reduce the use of antiparasitic drugs in swine farms.

## Figures and Tables

**Table 1:** Orthology descriptions of the top 30 genes that showed the highest *T. gondii* infection gene significance.

Protein	Gene Significance for *T. gondii* Infection	Total Quant Peptide Number	Description	Reference
GBP1*	0.82646008	307.2	Interferon induced guanylate binding protein 1. GBP1 contributes to anti-*T. gondii*.	[30,31]
TRIM21*	0.806586641	27.6	Tripartite motif containing 21. TRIM21-/- mice were highly susceptible to *T. gondii* infection and associated with higher *T. gondii* burden in the periphery and later in the brain.	[31]
ERAP1*	0.791323457	175.2	Endoplasmic reticulum aminopeptidase 1. Polymorphism of the human ERAP1 was significantly associate with susceptibility to human congenital toxoplasmosis (p<0.05).	[32,33]
C3*	0.789437052	648	Complement C3 precursor. C3 participates in complement-mediated killing of *T. gondii.*	[34]
C4*	0.777101166	126.25	Complement C4. Participating in antibody-dependent cytolysis of *T. gondii* requires C4.	[35]
GP91-PHOX*	0.768203441	57.5	Also known as NOX2. Mice deficient in NADPH oxidase isoform 2 (NOX2-/-) were not able to control *T. gondii* replication and produce IL-12p40, even upon LAM treatment.	[36]
GBP7*	0.761176836	63.6	Guanylate-binding protein 7. The reintroduction of Gbp7 into Gbpchr3-deleted cells partially restored the IFN-γ-dependent anti- *T. gondii* response.	[30]
STAT3*	0.758827825	145.2	Signal transducer and activator of transcription 3. STAT3 upregulates anti-inflammatory pathways to avoid over-inflammatory in *T. gondii* infected host.	[37]
CASP1*	0.754093682	26.66667	Caspase-1. Inflammasomes activate caspase-1, highly conserved Toxo1 haplotype directs resistance to toxoplasmosis and its associated caspase-1 dependent killing of parasite and host macrophage.	[38]
TAP1*	0.743196951	116.25	Antigen peptide transporter 1. TAP-1 regulates host ant- *T. gondii* infection by controlling NK cell IFN-γ production.	[39]
ITGAL*	0.799960205	87.6	Also known as CD11A, mediating natural killer cell mediated cytotoxicity. Expression of CD11a has been used as a marker of activation for the polyclonal antigen-specific T cell population in a variety of infectious settings, including toxoplasmosis.	[40,41]
HSP90AA1	0.854644744	1063.2	Also known as Hsp90. Hsp90 is an allosteric enhancer of iNOS which regulates nitric oxide biosynthetic process.	[42]
HSPA8	0.752183858	1106.4	Heat shock protein family A (Hsp70) member 8 (Also known as HSC70). The role of this gene in *T. gondii* infection is unclear. However, HSC70 from human 293T cells was shown to interact with a *T. gondii* protein designated *T. gondii* MAR domain-containing protein 4a (TgMCP4a).	[43]
STIP1	0.807262519	285.6	Stress induced phosphoprotein 1. Participating in protein correct folding.	NCBI annotation
PLCH1	0.854138738	21.25	Phospholipase C eta 1. Participating in inositol phosphate metabolism.	NCBI annotation
SERPINA3-3	0.853359004	272.4	SERPINA3-3 participates in innate immune system and platelet activation.	NCBI annotation
HSPA4	0.834185425	356.4	Heat shock protein family A (Hsp70) member 4. Participating in antigen processing and presentation.	NCBI annotation
HSPCB	0.815171015	538.8	Also known as Heat shock protein 90 alpha family class B member 1 (HSP90AB1). Participates in antigen processing and presentation, and protein processing in endoplasmic reticulum.	NCBI annotation
HYOU1	0.805621318	457.2	Hypoxia upregulated protein 1 (Also known as GRP170). Regulating protein processing in endoplasmic reticulum.	NCBI annotation
HSPA5	0.801396263	1140	Heat shock protein 5. (Also known as BIP). Participating in antigen presentation, protein folding, protein assembly and peptide loading of class I MHC.	NCBI annotation
PZP	0.797371798	489.6	PZP has endopeptidase inhibitor activity.	NCBI annotation
GVIN1	0.796028791	150	Interferon-induced very large GTPase 1. GTPase participates in anti-*T. gondii*. However, its role in *T. gondii* infection still unclear.	NCBI annotation
PDIA3	0.794989508	1144.8	Protein disulfide isomerase family A member 3. Participating in antigen presentation, protein folding, protein assemble and peptide loading of class I MHC	NCBI annotation
SLA-DRA	0.788350216	78	MHC class II DR-alpha. Participating in antigen processing and presentation.	NCBI annotation
LOC100513619	0.779559416	310.8	E3 ubiquitin-protein ligase RNF213-like.	NCBI annotation
HSPH1	0.764095739	325.2	Heat shock protein family H (Hsp110) member 1. Participating in protein processing in endoplasmic reticulum.	NCBI annotation
PLG	0.763548077	28.8	Plasminogen. Involved in complement and coagulation cascades.	NCBI annotation
HSP90B1	0.752494384	892.8	Heat shock protein 90 beta family member 1 participates in protein processing in endoplasmic reticulum.	NCBI annotation
PPIB	0.747401536	140.4	Peptidylprolyl isomerase B (PPIB) participates in extracellular matrix organization.	NCBI annotation
HSP70.2	0.743037358	236.4	Heat shock 70 kDa protein 1B. Participates in antigen processing and presentation, innate immune system and protein processing in endoplasmic reticulum.	NCBI annotation

Note: * indicates that the protecting role has been confirmed.

**Table 2:** Details of the potential anti-*T. gondii* proteins hotspots in chromosome 7.

Protein	Description	Chromosome Start	Chromosome End	Pathway	
SLA-1	MHC class I antigen 1	24748096	24751686	Antigen processing and presentation	NCBI annotation
C4	Complement C4A	27729074	27744209	Complement and coagulation cascades	NCBI annotation
CFB	Complement factor B	27771678	27777773	Complement and coagulation cascades	NCBI annotation
SLA-DRA	MHC class II DR-alpha	29036552	29042147	Antigen processing and presentation	NCBI annotation
SLA-DQB1	SLA-DQ beta1 domain	29180534	29188739	Antigen processing and presentation	NCBI annotation
TAP2	Transporter 2, ATP binding cassette subfamily B member	29256675	29268410	Antigen processing and presentation	NCBI annotation
TAP1	Transporter 1, ATP binding cassette subfamily B member	29274670	29283290	Antigen processing and presentation	NCBI annotation
LOC100520085	proteasome subunit beta type-9	29283635	29288788	Antigen processing-Cross presentation	NCBI annotation
TAPBP	TAP binding protein	34167262	34176573	Antigen processing and presentation	NCBI annotation
SERPINA3-3	SERPINA 3-3	122823963	123025049	Innate Immune System	NCBI annotation
LOC100153899	Serpin A3-8	122961721	122969848	Innate Immune System	NCBI annotation
LOC100156325	Serpin A3-6	123094660	123103381	Innate Immune System	NCBI annotation
SERPINA3-2	Alpha-1-antichymotrypsin 2	123110533	123119651	Innate Immune System	NCBI annotation
WARS	Tryptophanyl-tRNA synthetase	128677568	128702987	Tryptophanyl-tRNA synthetase	NCBI annotation
HSP90AA1	Heat shock protein 90 alpha family class A member 1	129758754	129760542	Regulation of eNOS activity	NCBI annotation

## Supplementary Materials

The following are available online at https://www.mdpi.com/2076-2607/8/4/518/s1, Supplementary Figure S1. Schematic illustration of CRISPR/Cas9-mediated knock-in DNA cassettes into the *H11* locus for expression of exogenous genes. Supplementary Figure S2. UpSet plot showing the sets of differentially expressed proteins from 10 different tissue samples, including the quantitative analysis of aggregate intersections between tissues. The vertical green bars show the number of intersecting proteins between tissues, denoted by the connected black circles below the histogram. The horizontal red bars show the protein set size. Supplementary Figure S3. PRM validation of the iTRAQ data. Supplementary Figure S4. Soft-power analysis of WGCNA. Supplementary Table S1. The primers used for the construction of the over-expressing cell lines. Supplementary Table S2. The summary of protein identification data. Supplementary Table S3. The repeatability analysis results. Supplementary File S1. The complete sequence map of the over-expression donor H11-cmv-c-HA plasmid. Supplementary File S2. GO and pathway enrichment analysis of the infected brain. Supplementary File S3. GO and pathway enrichment analysis of the infected liver. Supplementary File S4. GO and pathway enrichment analysis of the infected lung. Supplementary File S5. GO and pathway enrichment analysis of the infected spleen. Supplementary File S6. GO and pathway enrichment analysis of the infected mesenteric lymph nodes (MLNs). Supplementary File S7. List of the potential anti-*Toxoplasma gondii* proteins (PATPs).
