# Supplementary material for: iTRAQ-based Quantitative Proteomics Analysis Identifies Host Pathways Modulated during Toxoplasma gondii Infection in Swine
Source: Microorganisms. 2020 Apr 5;8(4):518. doi: 10.3390/microorganisms8040518 (PMC7232346; doi:10.3390/microorganisms8040518)
Supplement: Supplementary file 1 [file microorganisms-08-00518-s001.zip › supplementary materials/Supplemental Table S1.docx]

**Table S1. The primers used for the construction of over-expression cell lines.**

| Primer | Sequence | Product size |
| --- | --- | --- |
| HSP70.2-F | GCGGTGGCGGCCGCTCTAGGCCACCATGGCGAAGAGCGTGGCCATCGGCAT | 1,969bp |
| HSP70.2-R | ATCTGGAACATCGTATGGGTAATCCACCTCCTCGATGGTGGGGC |  |
| PDIA3-F | GCGGTGGCGGCCGCTCTAGGCCACCATGCGCCTCTGCCGCCTAGCGCT | 1,561bp |
| PDIA3-R | ATCTGGAACATCGTATGGGTAGAGATCCTCCTGTGCCTTCTTCT |  |
| Vector-F | TACCCATACGATGTTCCAGAT | 7,379bp |
| Vector-R | GGTGGCCTAGAGCGGCCGCCACCGC |  |
| Knock-in-F | CCATTGAGCCACGAACAGAACT | 1,878bp |
| Knock-in-R | CTTCACCAAGAGGTGACACCTTAAGG |  |

Note: Red sequences denote the vector adaptors used in the ligation of the coding sequence.
