# Supplementary material for: iTRAQ-based Quantitative Proteomics Analysis Identifies Host Pathways Modulated during Toxoplasma gondii Infection in Swine
Source: Microorganisms. 2020 Apr 5;8(4):518. doi: 10.3390/microorganisms8040518 (PMC7232346; doi:10.3390/microorganisms8040518)
Supplement: Supplementary file 1 [file microorganisms-08-00518-s001.zip › supplementary materials/Supplemental Table S2.docx]

**Table S2. The summary of protein identification data.**

| Tissues | Peptides | Unique peptides* | Proteins |
| --- | --- | --- | --- |
| Liver | 19,945 | 18,706 | 4,224 |
| Mesenteric lymph nodes | 23,262 | 22,043 | 5,103 |
| Spleen | 21,093 | 19,844 | 4,414 |
| Brain | 24,175 | 22,337 | 4,706 |
| Lung | 22,297 | 20,786 | 4,535 |

*Unique peptides represent peptides that were mapped to target proteins.
