# Supplementary material for: iTRAQ-based Quantitative Proteomics Analysis Identifies Host Pathways Modulated during Toxoplasma gondii Infection in Swine
Source: Microorganisms. 2020 Apr 5;8(4):518. doi: 10.3390/microorganisms8040518 (PMC7232346; doi:10.3390/microorganisms8040518)
Supplement: Supplementary file 1 [file microorganisms-08-00518-s001.zip › supplementary materials/Supplemental Table S3.docx]

**Table S3. The reproducibility of the proteomic results.**

| Infected tissues | CV* < 0.1 | CV < 0.2 | CV < 0.3 | CV < 0.4 | CV < 0.5 | CV < 0.6 | CV < 0.7 | CV < 0.8 | CV < 0.9 | CV < 1 | Mean CV |
| --- | --- | --- | --- | --- | --- | --- | --- | --- | --- | --- | --- |
| Liver 6 | 75.60% | 93.30% | 98.40% | 99.40% | 99.90% | 100.00% | 100.00% | 100.00% | 100.00% | 100.00% | 0.073 |
| Liver 18 | 63.40% | 88.50% | 96.70% | 99.30% | 99.80% | 100.00% | 100.00% | 100.00% | 100.00% | 100.00% | 0.096 |
| Mesenteric lymph nodes 6 | 74.20% | 93.50% | 98.10% | 99.40% | 99.80% | 99.90% | 99.90% | 100.00% | 100.00% | 100.00% | 0.075 |
| Mesenteric lymph nodes 18 | 63.80% | 88.70% | 96.50% | 99.00% | 99.80% | 100.00% | 100.00% | 100.00% | 100.00% | 100.00% | 0.096 |
| Spleen 6 | 72.70% | 92.40% | 98.20% | 99.50% | 99.70% | 100.00% | 100.00% | 100.00% | 100.00% | 100.00% | 0.078 |
| Spleen 18 | 61.00% | 87.00% | 96.00% | 99.00% | 99.00% | 100.00% | 100.00% | 100.00% | 100.00% | 100.00% | 0.1 |
| Brain 6 | 74.50% | 93.40% | 98.40% | 99.50% | 99.90% | 100.00% | 100.00% | 100.00% | 100.00% | 100.00% | 0.073 |
| Brain 18 | 57.00% | 85.00% | 94.00% | 98.00% | 99.00% | 100.00% | 100.00% | 100.00% | 100.00% | 100.00% | 0.11 |
| Lung 6 | 76.00% | 94.30% | 98.70% | 99.40% | 99.80% | 99.80% | 99.90% | 99.90% | 99.90% | 100.00% | 0.071 |
| Lung 18 | 64.40% | 88.50% | 96.50% | 98.80% | 99.70% | 100.00% | 100.00% | 100.00% | 100.00% | 100.00% | 0.095 |

*CV represents the coefficient of variations
