# Supplementary material for: iTRAQ-based Quantitative Proteomics Analysis Identifies Host Pathways Modulated during Toxoplasma gondii Infection in Swine
Source: Microorganisms. 2020 Apr 5;8(4):518. doi: 10.3390/microorganisms8040518 (PMC7232346; doi:10.3390/microorganisms8040518)
Supplement: Supplementary file 1 [file microorganisms-08-00518-s001.zip › supplementary materials/Supplemental file 1.docx]

**Supplemental file 1. The complete sequence map of the over-expression donor H11-cmv-c-HA plasmid.** Text highlighted in yellow represents NruI enzyme cutting site; in green represents H11 homologous arm F; in pink represents Puromycin resistance cassette; and in grey represents H11 homologous arm R. Nucleotide sequences shown in red color denote CMV promoter; in purple color denote multiple clone site; and in green color denote HA tag, poly A signal and transcription terminator.

TCGCGCGTTTCGGTGATGACGGTGAAAACCTCTGACACATGCAGCTCCCGGAGACGGTCACAGCTTGTCTGTAAGCGGATGCCGGGAGCAGACAAGCCCGTCAGGGCGCGTCAGCGGGTGTTGGCGGGTGTCGGGGCTGGCTTAACTATGCGGCATCAGAGCAGATTGTACTGAGAGTGCACCATATGCGGTGTGAAATACCGCACAGATGCGTAAGGAGAAAATACCGCATCAGGCGCCATTCGCCATTCAGGCTGCGCAACTGTTGGGAAGGGCGATCGGTGCGGGCCTCTTCGCTATTACGCCAGCTGGCGAAAGGGGGATGTGCTGCAAGGCGATTAAGTTGGGTAACGCCAGGGTTTTCCCAGTCACGACGTTGTAAAACGACGGCCAGTGAATTAGAACTCGGTACGCGCTGATCTTCCAGAGATTCGCGACCATTGAGCCACGAACAGAACTCCCTCTTACCAACTTATTACTACTAACTTCCCAAGTACTGGCTGCTCAGCTGCTTCCTTGGGCATGGGGGAGGGAGCACTATTTTTTCCTCTCCTGACTGCATCCTCTTCCTTTTAATTTCCATAAGGTTCCCTGTGGCCCTGTGCTTTTTTATTTTGAGGCCTTGCACATCCTTCTGGCCCTGATTGCTTCTCAACTCATCTTGTGCCTGCTGGACTTCCACCGTTGTTTCATGTATCTCGTTAGCTGAGATAGCACTTCCTCCTGCCCTTACCCTTTATCTGGCTCTTAGCTCCTAAAAACTGCATTATTAGCTTCCTCTTTTGCCTCTACTCTTACTCAACCAAAATTGTTTTAGACCTGTGGATCTAGCTTCTGCTGTGCTATTCTTAGGAACACTTTTATTTCCTCTTAGCTCCATCTCACCAGTTATTGGCTAATGGCTTTGCTTGGTACCTACATCTGTACATTTCTTTCGTACTAGCTTGTCGACTGAAAAAGGACTGTTGGTTCAACATGAAAGGGAAGGAGGTAAAAGAGGACACACAGGAAAGATGGATTGGGATTCAGGTCTCTGCTGTTGTTACTTGAGATTGCTTTCTAGTTCTACTTGTGGAAACAAAAAGCCTTTGCGAGATTCTAAACTGGAGTATTTCTGTAATTGAGGAGTCTTGCTCAGCAAATCCCACTTAGGGGACTAATGAAGTACCAGGAAGAGACAGACCATGCTCAATCCACAAAGCCAGGTTTTACTGAAATGTGACCTACTTTCTTATGTTCCTGGAAGTTTAGAGCGATCGCATGCATTAGTTATTAATAGTAATCAATTACGGGGTCATTAGTTCATAGCCCATATATGGAGTTCCGCGTTACATAACTTACGGTAAATGGCCCGCCTGGCTGACCGCCCAACGACCCCCGCCCATTGACGTCAATAATGACGTATGTTCCCATAGTAACGCCAATAGGGACTTTCCATTGACGTCAATGGGTGGAGTATTTACGGTAAACTGCCCACTTGGCAGTACATCAAGTGTATCATATGCCAAGTACGCCCCCTATTGACGTCAATGACGGTAAATGGCCCGCCTGGCATTATGCCCAGTACATGACCTTATGGGACTTTCCTACTTGGCAGTACATCTACGTATTAGTCATCGCTATTACCATGGTGATGCGGTTTTGGCAGTACATCAATGGGCGTGGATAGCGGTTTGACTCACGGGGATTTCCAAGTCTCCACCCCATTGACGTCAATGGGAGTTTGTTTTGGCACCAAAATCAACGGGACTTTCCAAAATGTCGTAACAACTCCGCCCCATTGACGCAAATGGGCGGTAGGCGTGTACGGTGGGAGGTCTATATAAGCAGAGCTGGTTTAGTGAACCGTCAGATCCGCTAGCGATTACGCCAAGCTCGAAATTAACCCTCACTAAAGGGAACAAAAGCTGGAGCTCCACCGCGGTGGCGGCCGCTCTAGGCCACCATGCCCGGGCGGATCCAAGCTTCTGCAGGAATTCGATATCGTCGACAGATCTCTCGAGTCTAGATACCCATACGATGTTCCAGATTACGCTTAAGGGCCCGGTACCTTAATTAATTAAGGTACCAGGTAAGTGTACCCAATTCGCCCTATAGTGAGTCGTATTACAATTCACTCGATCGCCCTTCCCAACAGTTGCGCAGCCTGAATGGCGAATGGAGATCCAATTTTTAAGTGTATAATGTGTTAAACTACTGATTCTAATTGTTTGTGTATTTTAGATTCACAGTCCCAAGGCTCATTTCAGGCCCCTCAGTCCTCACAGTCTGTTCATGATCATAATCAGCCATACCACATTTGTAGAGGTTTTACTTGCTTTAAAAAACCTCCCACACCTCCCCCTGAACCTGAAACATAAAATGAATGCAATTGTTGTTGTTAACTTGTTTATTGCAGCTTATAATGGTTACAAATAAAGCAATAGCATCACAAATTTCACAAATAAAGCATTTTTTTCACTGCATTCTAGTTGTGGTTTGTCCAAACTCATCAATGTATCTTAACGCGTgcgcagagcgcacatTgcccacagtccccgagaagttggggggaggggtcggcaattgatccggtgcctagagaaggtggcgcggggtaaactgggaaagtgatgtcgtgtactggctccgcctttttcccgagggtgggggagaaccgtatataagtgcagtagtcgccgtgaacgttctttttcgcaacgggtttgccgccagaacacaggaccggtTCTGGAgcgctgccaccATGGTGAGCAAGGGCGAGGAGCTGTTCACCGGGGTGGTGCCCATCCTGGTCGAGCTGGACGGCGACGTAAACGGCCACAAGTTCAGCGTGTCCGGCGAGGGCGAGGGAGATGCCACCTACGGCAAGCTGACCCTGAAGTTCATCTGCACCACCGGCAAGCTGCCCGTGCCCTGGCCCACCCTCGTGACCACCTTCGGCTACGGCGTGCAGTGCTTCGCCCGCTACCCCGACCACATGCGCCAGCACGACTTCTTCAAGTCCGCCATGCCCGAAGGCTACGTCCAGGAGCGCACCATCTTCTTCAAGGACGACGGCAACTACAAGACCCGCGCCGAGGTGAAGTTCGAGGGCGACACCCTGGTGAACCGCATCGAGCTGAAGGGCATCGACTTCAAGGAGGACGGCAACATCCTGGGGCACAAGCTGGAGTACAACTACAACAGCCACAACGTCTATATCATGGCCGACAAGCAGAAGAACGGCATCAAGGTGAACTTCAAGATCCGCCACAACATCGAGGACGGCAGCGTGCAGCTCGCCGACCACTACCAGCAGAACACCCCCATCGGCGACGGCCCCGTGCTGCTGCCCGACAACCACTACCTGAGCTACCAGTCCGCCCTGAGCAAAGACCCCAACGAGAAGCGCGATCACATGGTCCTGCTGGAGTTCGTGACCGCCGCCGGGATCACTCTCGGCATGGACGAGCTGTACAAGGGCGCAACAAACTTCTCTCTGCTGAAACAAGCCGGAGATGTCGAAGAGAATCCTGGACCGACCGAGTACAAGCCCACGGTGCGCCTCGCCACCCGCGACGACGTCCCCAGGGCCGTACGCACCCTCGCCGCCGCGTTCGCCGACTACCCCGCCACGCGCCACACCGTCGATCCGGACCGCCACATCGAGCGGGTCACCGAGCTGCAAGAACTCTTCCTCACGCGCGTCGGGCTCGACATCGGCAAGGTGTGGGTCGCGGACGACGGCGCCGCGGTGGCGGTCTGGACCACGCCGGAGAGCGTCGAAGCGGGGGCGGTGTTCGCCGAGATCGGCCCGCGCATGGCCGAGTTGAGCGGTTCCCGGCTGGCCGCGCAGCAACAGATGGAAGGCCTCCTGGCGCCGCACCGGCCCAAGGAGCCCGCGTGGTTCCTGGCCACCGTCGGCGTGTCGCCCGACCACCAGGGCAAGGGTCTGGGCAGCGCCGTCGTGCTCCCCGGAGTGGAGGCGGCCGAGCGCGCCGGGGTGCCCGCCTTCCTGGAGACCTCCGCGCCCCGCAACCTCCCCTTCTACGAGCGGCTCGGCTTCACCGTCACCGCCGACGTCGAGGTGCCCGAAGGACCGCGCACCTGGTGCATGACCCGCAAGCCCGGTGCCTGActgtgccttctagttgccagccatctgttgtttgcccctcccccgtgccttccttgaccctggaaggtgccactcccactgtcctttcctaataaaatgaggaaattgcatctcattaaagtctgagtaggtgtcattctattctggggggtggggtggggcaggacagcaagggggaggattgggaagacaatagcaggcatgctggggatgcggtgggctctattgGGCCGGCCTCAGGGTGGGCAGCTCTGGGTTTTATAGGCTACACTGTTAACACTCAGGCTGTTTTCTACCGTTTAGTCAAAATATAGTCACCTTGCCTGCTTCACCTGTCCATCAGAGAATGGCCTCATTAATTGACTCTCTAGTATGAAGTCAAAGTAGCTTTGGTGGCCCTAAATGGACAAGTATCAAGAGACTGGGTGAATTGAGGAGCTTGAGACTGTCACCTCAGATCGAAAAGACTGAAAAATCACCTCAGATCAAAAAGACTGAAAAATCTTCAGTCTGGAAAGGGGACTCAAAACCATAATTAGAGTATTCTGGTAGAATCCTTTTCTCCACTGTTATTCATACAGTTAAGGTGAATAACTAAAAGTAATTGTGAGCTGAGGAGTAAGATACAACACACAAGGAATCAGTTAACAGAGTCCGCGTGAAATTATAAATGGAAAGAATTATGACTTGAATCATAACTCTGAGGCCCCATTTTCCCTAACAACTTTTGTCCCAATAAACGTGGGTATTTGTTTGGGAGAAACTATCATATACATGATTACCCAGTAAACAGACTGTTTACTAAGTGGGTTTAATTTTAGAAATTGCGCGCTGCAATCTGGTATTAACCATACAACTACCTACCTATAGGGTCAGCCCAGCCTGAACTATCCCATTGGGGTCTTTATTAAGGCTCAAGAAACGGCCATAGCTTCTTCCTTTAAAATGAGTGTTTATTTCTATGAGCTTTAAAGAAAAAAACAGATAATTTCCCTCAACCTACTGAAGAGGAAGGGATTCAGGAAGAAATAAACACAACAATGCCATTCACTTCATCGCGAAATCGTAGAACTGCAGGCGTGCAAACTTGGCGTAATCATGGTCATAGCTGTTTCCTGTGTGAAATTGTTATCCGCTCACAATTCCACACAACATACGAGCCGGAAGCATAAAGTGTAAAGCCTGGGGTGCCTAATGAGTGAGCTAACTCACATTAATTGCGTTGCGCTCACTGCCCGCTTTCCAGTCGGGAAACCTGTCGTGCCAGCTGCATTAATGAATCGGCCAACGCGCGGGGAGAGGCGGTTTGCGTATTGGGCGCTCTTCCGCTTCCTCGCTCACTGACTCGCTGCGCTCGGTCGTTCGGCTGCGGCGAGCGGTATCAGCTCACTCAAAGGCGGTAATACGGTTATCCACAGAATCAGGGGATAACGCAGGAAAGAACATGTGAGCAAAAGGCCAGCAAAAGGCCAGGAACCGTAAAAAGGCCGCGTTGCTGGCGTTTTTCCATAGGCTCCGCCCCCCTGACGAGCATCACAAAAATCGACGCTCAAGTCAGAGGTGGCGAAACCCGACAGGACTATAAAGATACCAGGCGTTTCCCCCTGGAAGCTCCCTCGTGCGCTCTCCTGTTCCGACCCTGCCGCTTACCGGATACCTGTCCGCCTTTCTCCCTTCGGGAAGCGTGGCGCTTTCTCATAGCTCACGCTGTAGGTATCTCAGTTCGGTGTAGGTCGTTCGCTCCAAGCTGGGCTGTGTGCACGAACCCCCCGTTCAGCCCGACCGCTGCGCCTTATCCGGTAACTATCGTCTTGAGTCCAACCCGGTAAGACACGACTTATCGCCACTGGCAGCAGCCACTGGTAACAGGATTAGCAGAGCGAGGTATGTAGGCGGTGCTACAGAGTTCTTGAAGTGGTGGCCTAACTACGGCTACACTAGAAGAACAGTATTTGGTATCTGCGCTCTGCTGAAGCCAGTTACCTTCGGAAAAAGAGTTGGTAGCTCTTGATCCGGCAAACAAACCACCGCTGGTAGCGGTGGTTTTTTTGTTTGCAAGCAGCAGATTACGCGCAGAAAAAAAGGATCTCAAGAAGATCCTTTGATCTTTTCTACGGGGTCTGACGCTCAGTGGAACGAAAACTCACGTTAAGGGATTTTGGTCATGAGATTATCAAAAAGGATCTTCACCTAGATCCTTTTAAATTAAAAATGAAGTTTTAAATCAATCTAAAGTATATATGAGTAAACTTGGTCTGACAGTTACCAATGCTTAATCAGTGAGGCACCTATCTCAGCGATCTGTCTATTTCGTTCATCCATAGTTGCCTGACTCCCCGTCGTGTAGATAACTACGATACGGGAGGGCTTACCATCTGGCCCCAGTGCTGCAATGATACCGCGAGACCCACGCTCACCGGCTCCAGATTTATCAGCAATAAACCAGCCAGCCGGAAGGGCCGAGCGCAGAAGTGGTCCTGCAACTTTATCCGCCTCCATCCAGTCTATTAATTGTTGCCGGGAAGCTAGAGTAAGTAGTTCGCCAGTTAATAGTTTGCGCAACGTTGTTGCCATTGCTACAGGCATCGTGGTGTCACGCTCGTCGTTTGGTATGGCTTCATTCAGCTCCGGTTCCCAACGATCAAGGCGAGTTACATGATCCCCCATGTTGTGCAAAAAAGCGGTTAGCTCCTTCGGTCCTCCGATCGTTGTCAGAAGTAAGTTGGCCGCAGTGTTATCACTCATGGTTATGGCAGCACTGCATAATTCTCTTACTGTCATGCCATCCGTAAGATGCTTTTCTGTGACTGGTGAGTACTCAACCAAGTCATTCTGAGAATAGTGTATGCGGCGACCGAGTTGCTCTTGCCCGGCGTCAATACGGGATAATACCGCGCCACATAGCAGAACTTTAAAAGTGCTCATCATTGGAAAACGTTCTTCGGGGCGAAAACTCTCAAGGATCTTACCGCTGTTGAGATCCAGTTCGATGTAACCCACTCGTGCACCCAACTGATCTTCAGCATCTTTTACTTTCACCAGCGTTTCTGGGTGAGCAAAAACAGGAAGGCAAAATGCCGCAAAAAAGGGAATAAGGGCGACACGGAAATGTTGAATACTCATACTCTTCCTTTTTCAATATTATTGAAGCATTTATCAGGGTTATTGTCTCATGAGCGGATACATATTTGAATGTATTTAGAAAAATAAACAAATGGGGGTTCCGCGCACATTTCCCCGAAAAGTGCCACCTGACGTCTAAGAAACCATTATTATCATGACATTAACCTATAAAAATAGGCGTATCACGAGGCCCTTTCGTC
