# Supplementary figures and images for: iTRAQ-based Quantitative Proteomics Analysis Identifies Host Pathways Modulated during Toxoplasma gondii Infection in Swine
Source: Microorganisms. 2020 Apr 5;8(4):518. doi: 10.3390/microorganisms8040518 (PMC7232346; doi:10.3390/microorganisms8040518)

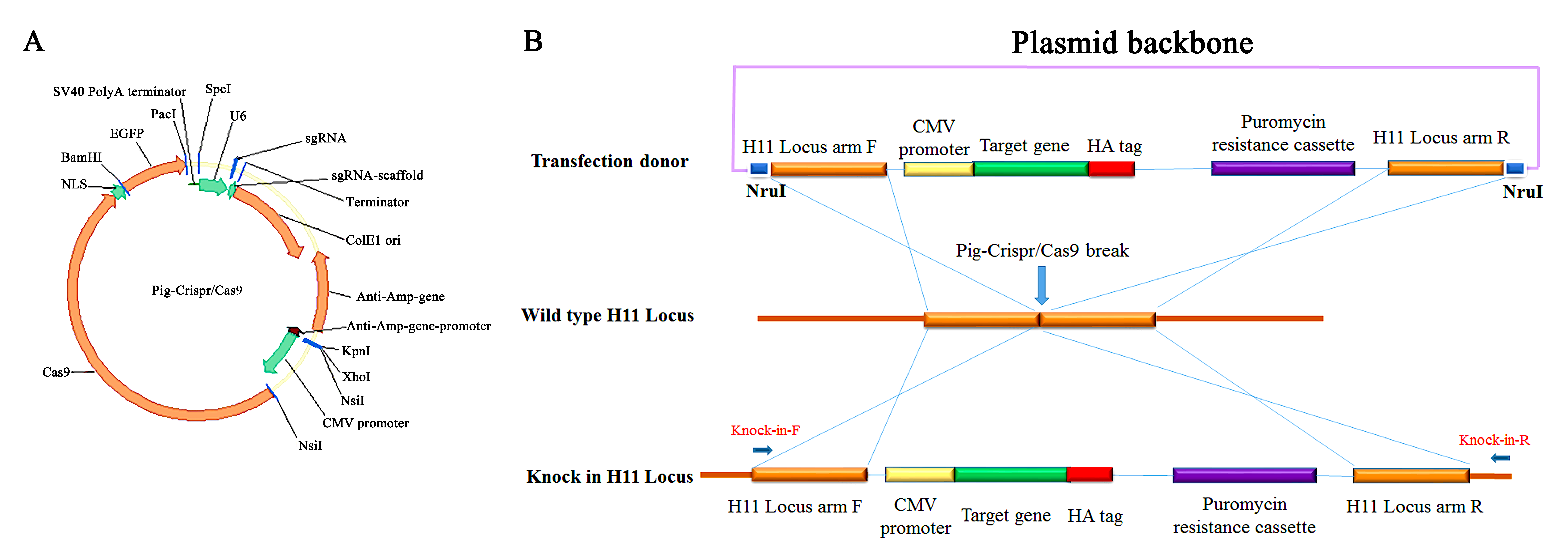

Supplement: Supplementary file 1 [file microorganisms-08-00518-s001.zip › supplementary materials/Supplemental Figure S1.tif]

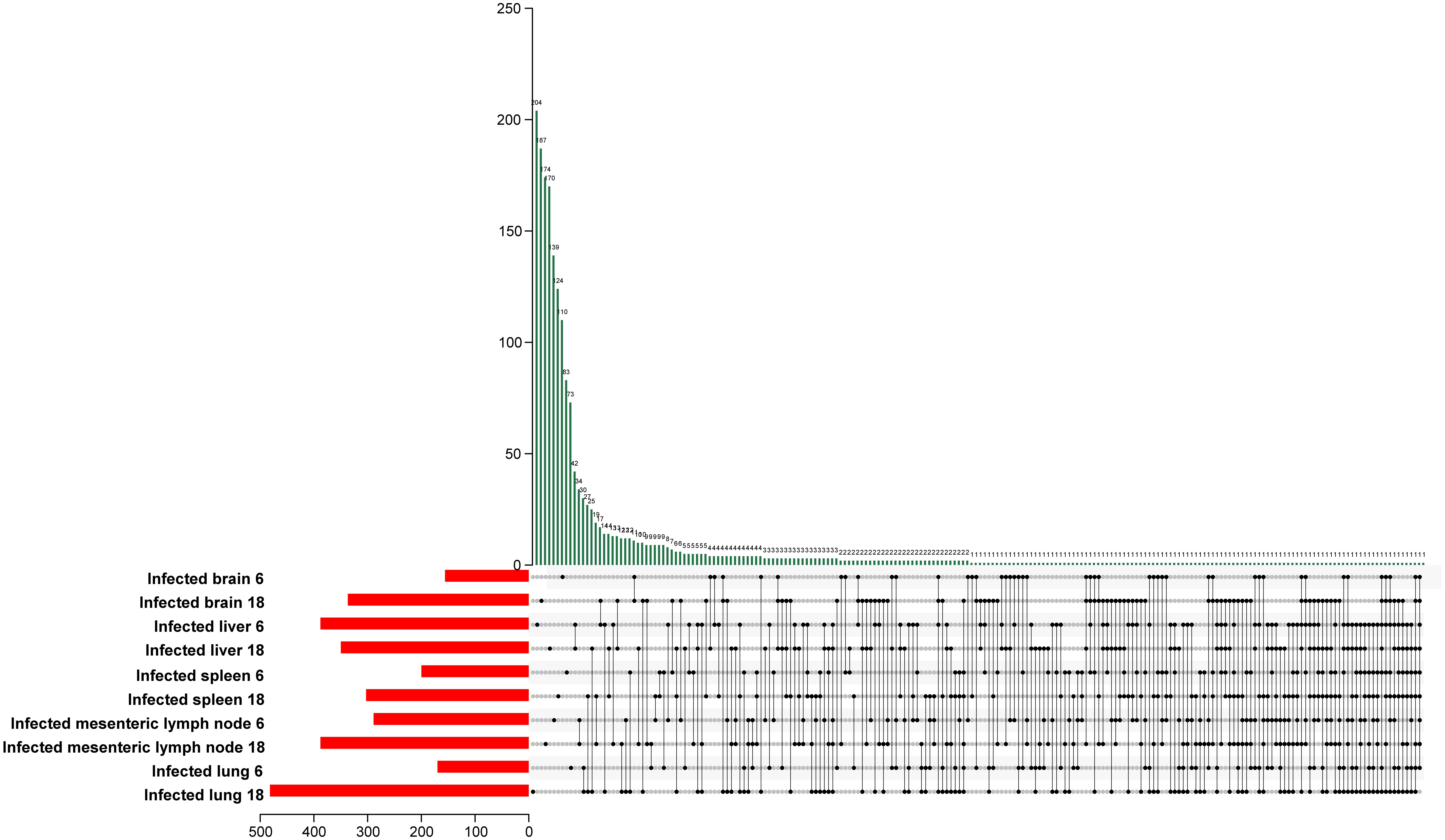

Supplement: Supplementary file 1 [file microorganisms-08-00518-s001.zip › supplementary materials/Supplemental Figure S2.jpg]

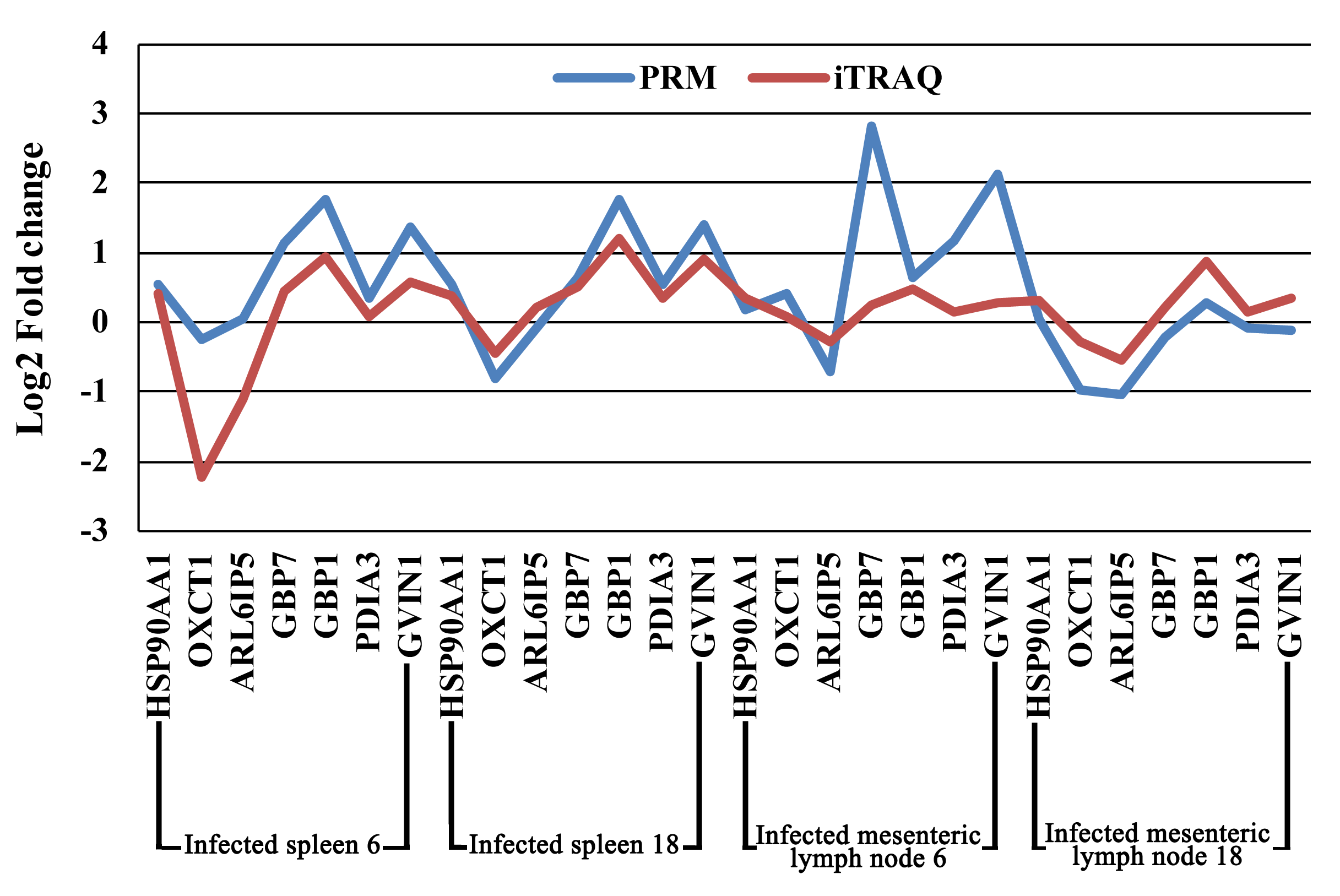

Supplement: Supplementary file 1 [file microorganisms-08-00518-s001.zip › supplementary materials/Supplemental Figure S3.tif]

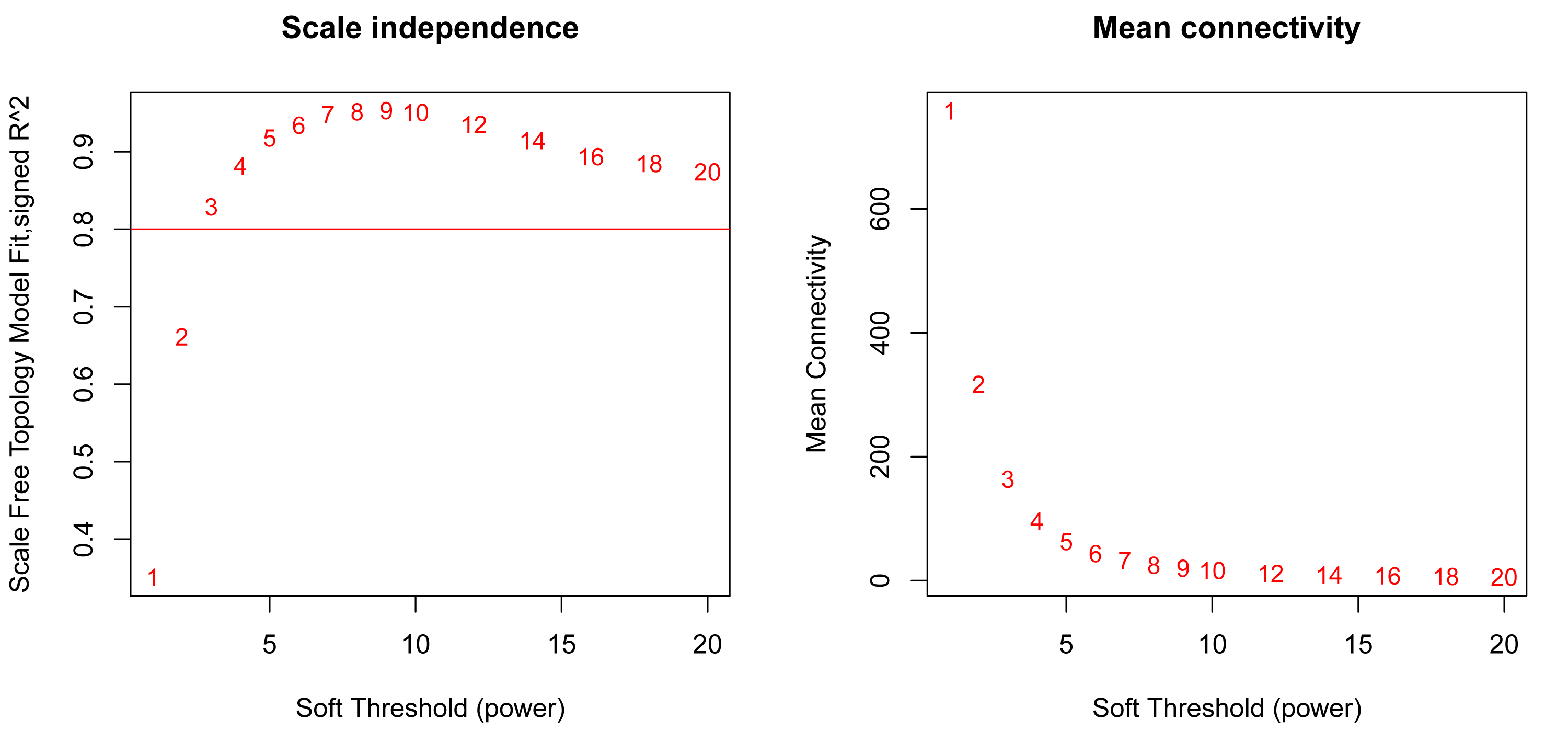

Supplement: Supplementary file 1 [file microorganisms-08-00518-s001.zip › supplementary materials/Supplemental Figure S4.tif]
